# Supplementary material for: Ocean driven retreat of the Northeast Greenland Ice Stream following the Last Glacial Maximum
Source: Nat Commun. 2025 Nov 29;16:10961. doi: 10.1038/s41467-025-66671-2 (PMC12689639; doi:10.1038/s41467-025-66671-2)
Supplement: Supplementary file 1 — Supplementary Information [file 41467_2025_66671_MOESM1_ESM.pdf]

Supplementary information

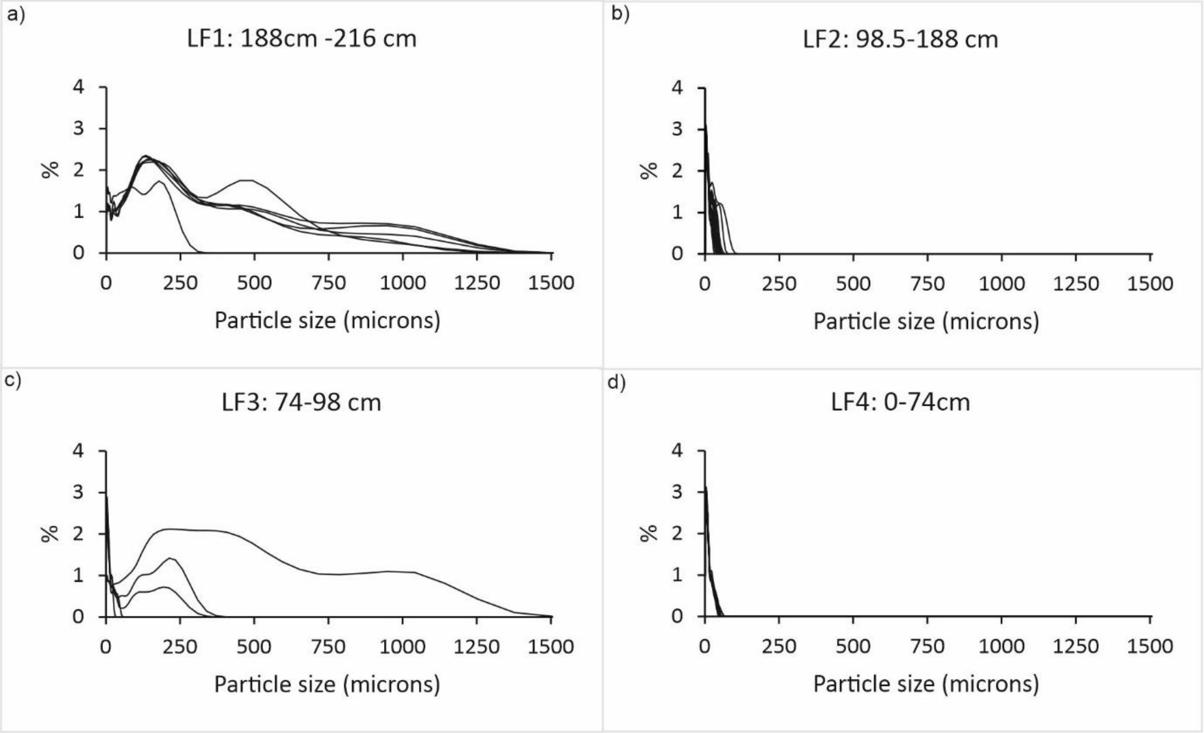

Supplementary Figure 1: Particle size distribution in microns for the different lithofacies in core 144GC. Particle size distributions for samples in a) Lithofacies 1 (LF1), the diamicton (Dmm) between core depths 188-216 cm, b) Lithofacies 2(LF2) the laminated mud (Fl) between core depths 188-98.5 cm, c) Lithofacies 3 (LF3) the massive mud with dropstones (Fmd) between core depths 74-98.5 cm and d) Lithofacies 4 (LF4) the massive mud (Fm) between core depths 0-74 cm.

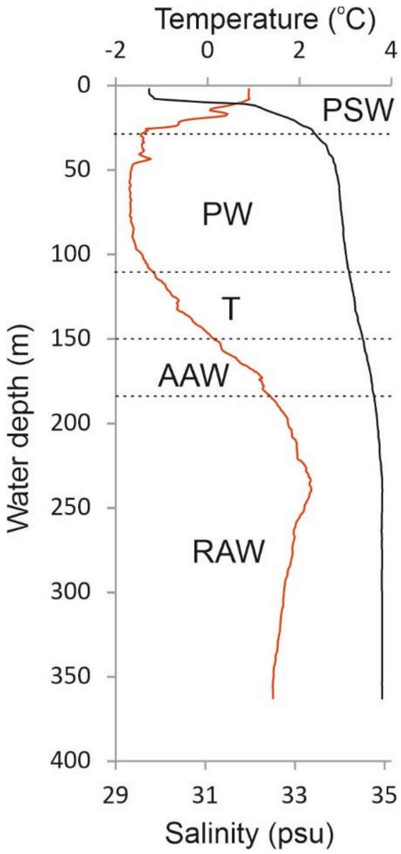

Supplementary Figure 2: Conductivity, temperature and depth (CTD) profile from core site 144GC. The figure plots the change in temperature in Celsius (°C) and salinity in practical salinity units (PSU) with water depth in meters. PSW: Polar Surface Water; PW: Polar Water; T: Transition; AAW: Arctic Atlantic Water; RAW: Return Atlantic Water.

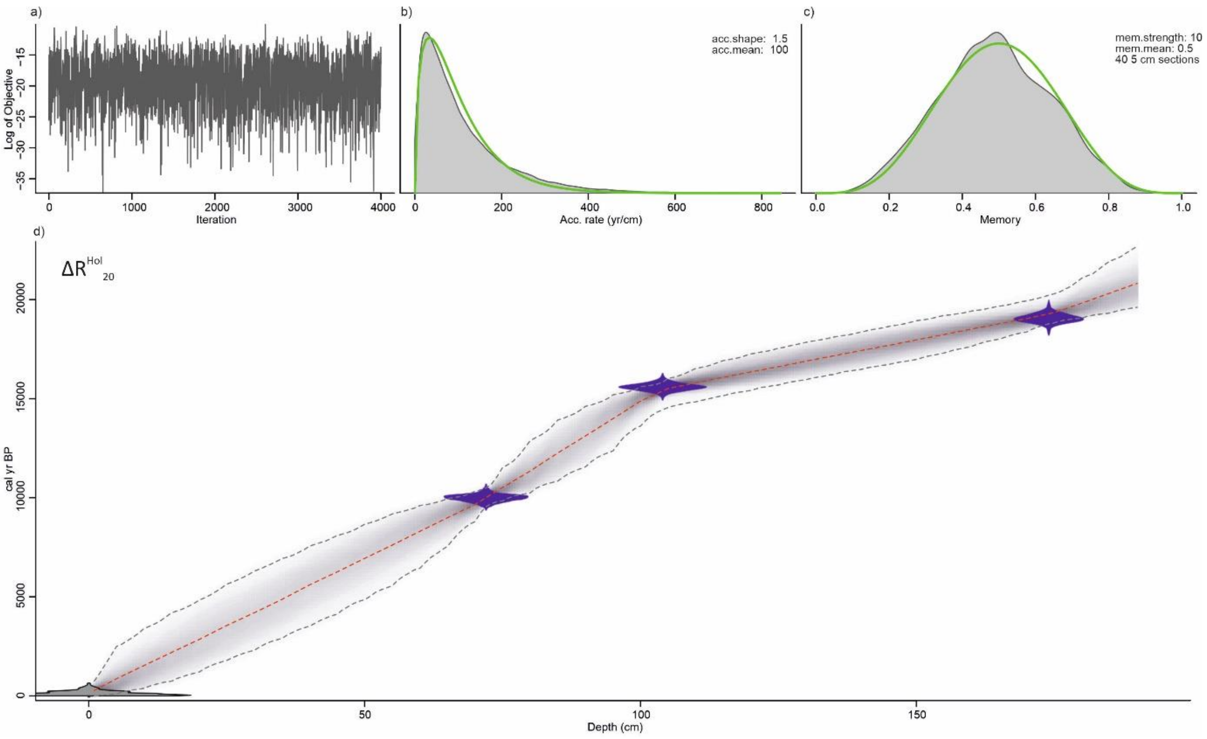

Supplementary Figure 3: Age- depth models for core 144GC using the local Holocene reservoir correction (ΔR^{Hol}_{20}) calibration. a) depict the Markov chain Monte Carlo (MCMC) iterations; b) the prior (green curve) and posterior (grey histogram) distributions for the accumulation rate; c) the prior (green) and posterior (grey histogram) of the memory; d) the age-depth model with the calibrated radiocarbon age in blue, the mean modelled age in red dashed line and the 95% confidence range in the grey envelope. The calibrated age at 1 cm (grey with black outline) is an assumed core top age of 1 yr BP and is used to constrain the Holocene section of the chronology. This section of the core was not used to make comparisons with other published proxy reconstructions shown in Figure 5. The age-depth models were generate using RBacon[1].

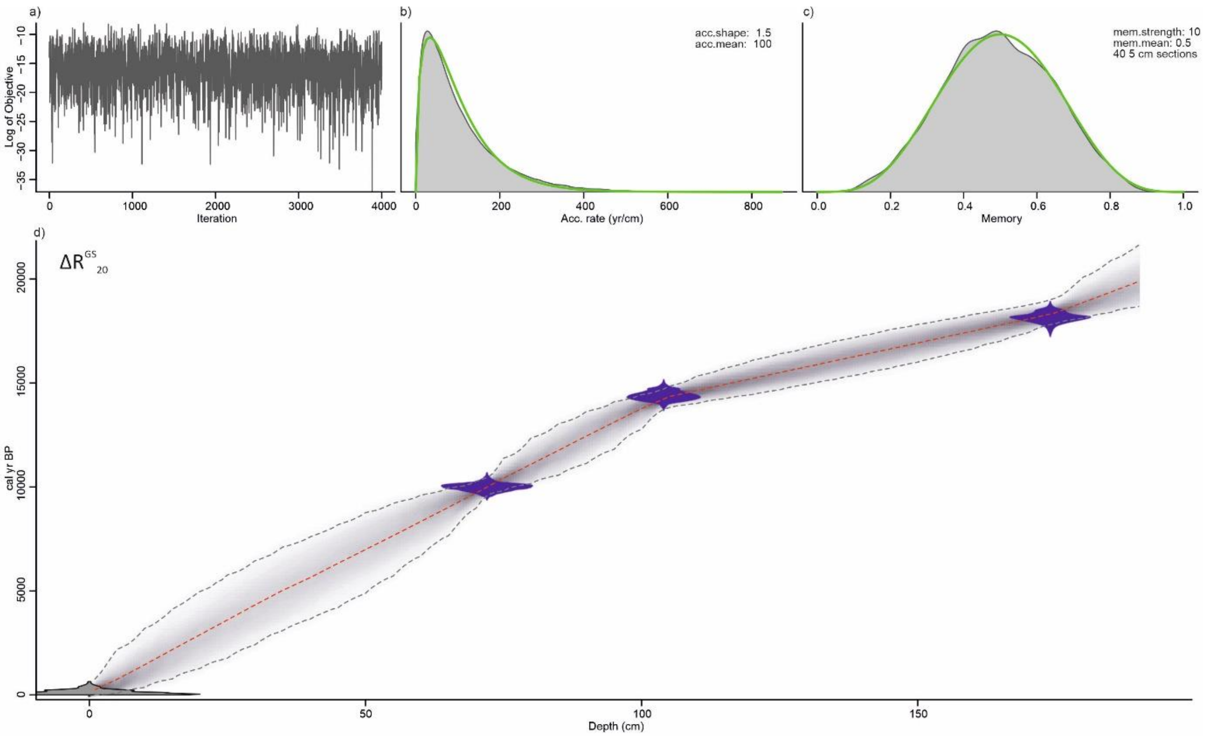

Supplementary Figure 4: Age- depth models for core 144GC using the local Glacial reservoir ($\Delta R^{GS}{}_{20}$) calibration. a) depict the Markov chain Monte Carlo (MCMC) iterations; b) the prior (green curve) and posterior (grey histogram) distributions for the accumulation rate; c) the prior (green) and posterior (grey histogram) of the memory; d) the age-depth model with the calibrated radiocarbon age in blue, the mean modelled age in red dashed line and the 95% confidence range in the grey envelope. The calibrated age at 1 cm (grey with black outline) is an assumed core top age of 1 yr BP and is used to constrain the Holocene section of the chronology. This section of the core was not used to make comparisons with other published proxy reconstructions shown in Figure 5. The age-depth models were generate using RBacon[1].

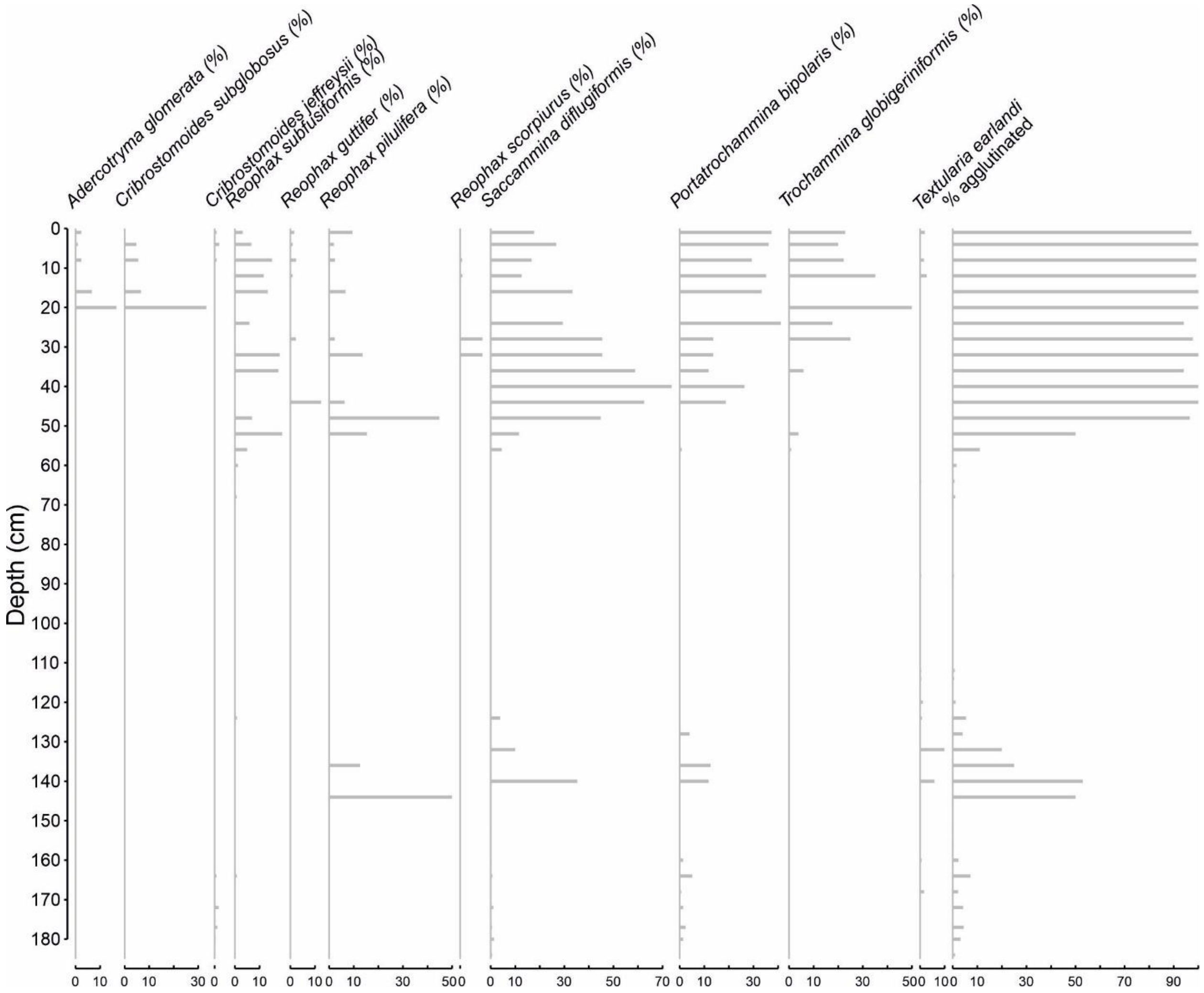

Supplementary Figure 5: Agglutinated foraminifera assemblage plotted against depth. The figure plots the relative abundance in % for each agglutinated species identified in the core plotted against depth and the proportion of the benthic count in % that consist of agglutinated species.

Supplementary information references

Blaauw, M & J. Andrés Christen, J.R. Flexible paleoclimate age-depth models using an autoregressive gamma process. *Bayesian Analysis* **6(3)**, 457-474 (2011).
